# Dysmenorrhœa

**Published:** 1882-11

**Authors:** E. G. Cook


### DYSMENORRHŒA.

The patient, Mrs. E. D., æt. 36, is married twelve years, sterile, and greatly desiring children. I saw her first in December, 1879. She was suffering agonies from an ineffectual attempt to menstruate. For the last five or six years the monthly flow had been growing less, until one small napkin was all that was needed. The pain in the meantime had increased to an alarming degree. She had grown very stout, as most such cases do, and had fearful congestive headaches during the week of menstruation, and terrible sick headaches once in eight or ten days in the interim. I found a sharp retroflexion, the fundus lower in the pelvis than the os uteri, and the whole organ very much enlarged, hard, and extremely sensitive to the touch. I introduced bent carbolized sponge tents daily until I could straighten the organ, and lift it to its natural position. Then a galvanic stem was used, which caused a flow for six weeks, more or less ; the pessary was removed and cleansed once in five or six days, and the oxide of zinc scraped carefully from it. (The uterine acid is the cause of this oxidation, and ordinarily keeps the copper portion bright. It sometimes, however, becomes an irritant, and is then changed for a hard rubber stem). Her head symptoms improved at once. The sick headaches recurred at longer intervals, were less severe, and finally ceased altogether. She has since worn either the galvanic or rubber stem (two years and three months) until April last. I removed it, and pregnancy followed. During the last eighteen months her health has been perfect, the flow free and painless. Her obesity is much reduced. Remedies were administered during the first year only. At this writing she is four months and a half advanced in pregnancy.—*Mrs. E. G. Cook, M. D., in N. Y. Med. Journal.*

